# The role of biocompatible coatings of biomaterials for creation of direct and appropriate chemical bounding between bioimplant and bone tissue

**Published:** 2012-11

**Authors:** Touraj Shirzadian, Seyedreza Bagheri, Hamidreza SaeidiBorojeni, Parviz Ghaffari, Fezollah Foroughi, Mohammad Mahboubi

**Affiliations:** ^*a*^Vice Chancellery for Research and Technology, Kermanshah University of Medical Sciences, Kermanshah, Iran.; ^*b*^Department of Neurosurgical, Kermanshah University of Medical Sciences, Kermanshah, Iran.; ^*c*^Department of Orthopedic Surgery, Kermanshah University of Medical Sciences, Kermanshah, Iran.

## Abstract

**Background:**

Nowadays, the surface modification of biomaterials to increase biocompatibility and improve other aspects of environmental performance is widely prevalent and is developing. Biological host response depends on the primary interactions of biological and biomaterials systems at the molecular surfaces. Therefore, the surface properties at the atomic scale influence on compatibility and optimal performance of the material in body. The present study aims to survey the most common surface modification techniques of biomaterials focusing on the surface coating techniques and their applications in bone tissue engineering and tissue repairing field. Furthermore, the process and features of biomaterial surface coating in complex superficial modification of biomaterials as a representation of biomaterials interfaces with the biological environment are discussed. Finally, the potential applications and advantages of these techniques in repairing damaged tissues in neurosurgery and orthopedic surgery are presented.

**Methods:**

In this review article, the most common and important methods of surface modification of biomaterials (thermal spray, electrophoresis, pulsed laser deposition, electrochemical, biomimetic, sputtering, Chemical Vapor Deposition (CVD), and, Sol–Gel techniques) which the reputable manufacture companies of biomaterial are applying and many published articles in the biomaterial field (since 2004 up to now). Furthermore, the potential applications and current positions of these methods in bone tissue engineering and effective factors for an optimal tissue repairing using biomaterial surface coating are presented.

**Results:**

The Sol-gel method is suitable for obtaining nanoscale structures. Plasma spraying method has a high-speed balance and is the only commercially available method. The pulsed laser coating method can be used in multilayer coatings and structures with a fixed Stoichiometric.

The electrophoresis coating methods are able to coat relatively complex shapes with precise control over coating thickness.In recent years, several coating methods such as electrochemical, biomimetic, sputtering, and CVD have been proposed and used by researchers some of them with good satisfactory results.

**Conclusions:**

Findings of our survey show that surface modification of biomaterials can keep the crucial physical properties of the biomaterials unchanged which is an important feature in biological reactions. The main advantages of biomaterial surface modification including surface coating can be presented as follows:

**Reducing protein adsorption:**Biomaterials sometimes need to have the lowest protein adsorption, otherwise, will cause uncontrolled immune responses.

**Lack of cellular coherency**

**Cellular absorption:**Biomaterials that are used as replacement tissue cells must have high capability for cell absorption and also should facilitate their growth process.

**Reducing Clot formation:**Biomaterials that are used as blood implants should have the lowest capacity of clot formation.

**High coherency to bacteria **

**Reducing friction and grinding**

**Keywords:**

Biomaterials, Coating, Biocompatibility, Grinding, Bounding, Implant


					Volume 4, Suppl. 1 Nov 2012
Publisher: Kermanshah University of Medical Sciences
URL: http://www.jivresearch.org
Copyright:
Congress of Iranian Neurosurgeons, 14-16 November 2012, Kermanshah, Iran
Paper No. 39
The role of biocompatible coatings of biomaterials for
creation of direct and appropriate chemical bounding
between bioimplant and bone tissue
Touraj Shirzadian a,*
, Seyedreza Bagheri b
, Hamidreza SaeidiBorojeni b
, Parviz Ghaffari c
,
Fezollah Foroughi a
, Mohammad Mahboubi a
a
Vice Chancellery for Research and Technology, Kermanshah University of Medical Sciences, Kermanshah, Iran.
b
Department of Neurosurgical, Kermanshah University of Medical Sciences, Kermanshah, Iran.
c
Department of Orthopedic Surgery, Kermanshah University of Medical Sciences, Kermanshah, Iran.
Abstract:
Background: Nowadays, the surface modification of biomaterials to increase biocompatibility and improve other
aspects of environmental performance is widely prevalent and is developing. Biological host response depends on
the primary interactions of biological and biomaterials systems at the molecular surfaces. Therefore, the surface
properties at the atomic scale influence on compatibility and optimal performance of the material in body. The
present study aims to survey the most common surface modification techniques of biomaterials focusing on the surface
coating techniques and their applications in bone tissue engineering and tissue repairing field. Furthermore, the
process and features of biomaterial surface coating in complex superficial modification of biomaterials as a
representation of biomaterials interfaces with the biological environment are discussed. Finally, the potential
applications and advantages of these techniques in repairing damaged tissues in neurosurgery and orthopedic
surgery are presented.
Methods: In this review article, the most common and important methods of surface modification of biomaterials
(thermal spray, electrophoresis, pulsed laser deposition, electrochemical, biomimetic, sputtering, Chemical Vapor
Deposition (CVD), and, Sol-Gel techniques) which the reputable manufacture companies of biomaterial are applying
and many published articles in the biomaterial field (since 2004 up to now). Furthermore, the potential applications
and current positions of these methods in bone tissue engineering and effective factors for an optimal tissue repairing
using biomaterial surface coating are presented.
Results: The Sol-gel method is suitable for obtaining nanoscale structures. Plasma spraying method has a high-speed
balance and is the only commercially available method. The pulsed laser coating method can be used in multilayer
coatings and structures with a fixed Stoichiometric.
The electrophoresis coating methods are able to coat relatively complex shapes with precise control over coating
thickness.In recent years, several coating methods such as electrochemical, biomimetic, sputtering, and CVD have been
proposed and used by researchers some of them with good satisfactory results.
Conclusions: Findings of our survey show that surface modification of biomaterials can keep the crucial physical
properties of the biomaterials unchanged which is an important feature in biological reactions. The main advantages
of biomaterial surface modification including surface coating can be presented as follows:
Reducing protein adsorption: Biomaterials sometimes need to have the lowest protein adsorption, otherwise, will
cause uncontrolled immune responses.
Lack of cellular coherency
Cellular absorption: Biomaterials that are used as replacement tissue cells must have high capability for cell
absorption and also should facilitate their growth process.
Volume 4, Suppl. 1 Nov 2012
Publisher: Kermanshah University of Medical Sciences
URL: http://www.jivresearch.org
Copyright:
Congress of Iranian Neurosurgeons, 14-16 November 2012, Kermanshah, Iran
Reducing Clot formation: Biomaterials that are used as blood implants should have the lowest capacity of clot
formation.
High coherency to bacteria
Reducing friction and grinding
Keywords:
Biomaterials , Coating , Biocompatibility, Grinding , Bounding
* Corresponding Author at:
Touraj Shirzadian: Vice Chancellery for Research and Technology, Kermanshah University of Medical Sciences, Kermanshah, Iran. Tel: 09183366806,
Email: t.shirzadian@kums.ac.ir, (Shirzadian T.).

				

